# Frequency of Epstein - Barr Virus in Patients Presenting with Acute Febrile Illness in Kenya

**DOI:** 10.1371/journal.pone.0155308

**Published:** 2016-05-10

**Authors:** Clement Masakhwe, Horace Ochanda, Nancy Nyakoe, Daniel Ochiel, John Waitumbi

**Affiliations:** 1 School of Biological Sciences, University of Nairobi, Nairobi, Kenya; 2 Walter Reed Project/Kenya Medical Research Institute, Kisumu, Kenya; University of British Columbia, CANADA

## Abstract

**Background:**

Most acute febrile illnesses (AFI) are usually not associated with a specific diagnosis because of limitations of available diagnostics. This study reports on the frequency of EBV viremia and viral load in children and adults presenting with febrile illness in hospitals in Kenya.

**Methodology/Principal Findings:**

A pathogen surveillance study was conducted on patients presenting with AFI (N = 796) at outpatient departments in 8 hospitals located in diverse regions of Kenya. Enrollment criterion to the study was fever without a readily diagnosable infection. All the patients had AFI not attributable to the common causes of fever in Kenyan hospitals, such as malaria or rickettsiae, leptospira, brucella and salmonella and they were hence categorized as having AFI of unknown etiology. EBV was detected in blood using quantitative TaqMan-based qPCR targeting a highly conserved BALF5 gene. The overall frequency of EBV viremia in this population was 29.2%, with significantly higher proportion in younger children of <5years (33.8%, p = 0.039) compared to patients aged ≥5 years (26.3% for 5–15 years or 18.8% for >15 years). With respect to geographical localities, the frequency of EBV viremia was higher in the Lake Victoria region (36.4%), compared to Kisii highland (24.6%), Coastal region (22.2%) and Semi-Arid region (25%). Furthermore, patients from the malaria endemic coastal region and the Lake Victoria region presented with significantly higher viremia than individuals from other regions of Kenya.

**Conclusions/Significance:**

This study provides profiles of EBV in patients with AFI from diverse eco-regions of Kenya. Of significant interest is the high frequency of EBV viremia in younger children. The observed high frequencies of EBV viremia and elevated viral loads in residents of high malaria transmission areas are probably related to malaria induced immune activation and resultant expansion of EBV infected B-cells.

## Introduction

Acute febrile illnesses (AFI), defined as non-specific illnesses presenting with fever ≥ 38°C lasting for less than two weeks without a readily diagnosable source after routine clinical evaluation [1,2], are the most common causes of outpatient attendance and mortality, especially among children [3,4]. Studies that have attempted to identify pathogens associated with AFI report of several etiologies [2,4–8].

In malaria endemic regions, AFI, especially in children are clinically managed as malaria even in the absence of parasites [7–9]. This often leads to over diagnosis of malaria [3,4,9,10]. As the global incidence of malaria declines [11], a better understanding of causes of non-malaria fever will be required to guide effective clinical management.

EBV is reported to account for a significant occurrence of AFI cases [2,8]. Symptomatic EBV is commonly characterized by febrile episodes, and has many clinical signs that cannot be differentiated from those of other febrile illness [12–15]. Primary EBV infection can also result in a wide variety of benign and neoplastic diseases such as Hodgkin’s lymphoma, Burkitt’s lymphoma, post-transplant lymphoproliferative disorders (PTLDs), and oral hairy leukoplakia in AIDS patients [14,16].

EBV infection is common, with approximately 95% of the global adult population showing serological evidence of exposure [14,15]. In Kenya, the virus is also strongly associated with occurrence of endemic Burkitt’s lymphoma in malaria holoendemic regions [17–19]. The extent to which EBV is associated with non-malaria febrile illness remains undefined. This is partly due to the challenges in the definitive diagnosis of EBV infection. Clinically, ELISA is routinely used for the diagnosis of acute EBV infection. However, this technique has inherent limitations [20]. Firstly, the assay is less sensitive in immunocompromised patients. Further, since there is a window period in which antibodies are not produced, false negative results are possible [20–22]. The limitations of ELISA technique for EBV diagnosis have, to a large extent, been minimized with the use of real time qPCR [23]. The latter technique is more sensitive, especially in the acute phase of EBV infection [23] and can be used to quantitate EBV viral load [21,24–28]. Nevertheless, EBV antibody tests have many other attributes over PCR, especially in helping to classify EBV status. A past infection is confirmed by presence of VCA IgG and EBNA-1 IgG, in the absence of VCA IgM [29,30]. As reported in these studies, a patient is considered susceptible if he/she is negative for VCA IgG, VCA IgM, and EBNA-1 IgG. If tests for VCA IgM and VCA IgG are positive and those for EBNA-1 IgG are negative, a patient is considered to have had a primary or acute infection [29].

The current study investigated the frequency of EBV viremia and viral load in patients with non-malaria febrile illness residing in different eco-regions of Kenya.

## Methods

### Ethical approval

Eligible subjects were recruited under a study protocol that was approved by the Ethical Review Committee of the Kenya Medical Research Institute (SSC #, 1282) and the Walter Reed Army Institute of Research Human Subject Protection Board in the United States of America (WRAIR HSPB # 1402).

### Study design

**Fig 1:**
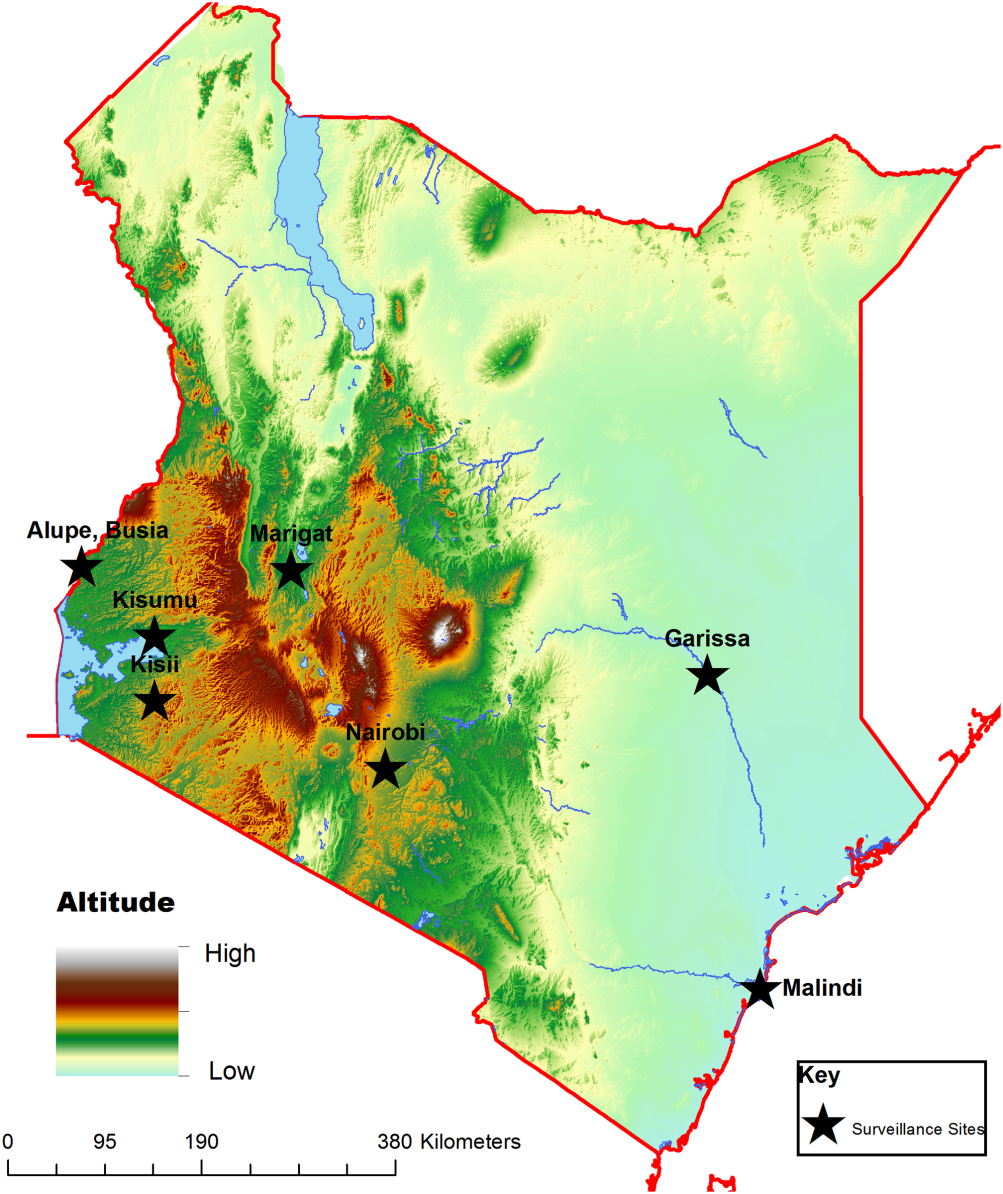
Map of Kenya showing the locations of various surveillance hospitals. Lake Victoria basin: Kisumu District Hospital, New Nyanza Provincial Hospital and Alupe District Hospital. Kisii highland: Kisii District Hospital. Semi-arid: Marigat District Hospital on floor of the Rift Valley. Arid north eastern Kenya: Garissa District Hospital and Iftin sub-District Hospital. Coast of Indian Ocean: Malindi District Hospital. The map was generated in house using the ArcView^®^10.0 application (Environmental Systems Research Institute, Redlands, CA, USA). Elevation base data was downloaded from the world resources institute website (http://www.wri.org/).

The study assessed patients presenting with febrile illness in 8 hospitals in Kenya for infections with EBV (Fig 1). Recruitment was open to patients one year of age or older presenting as outpatients at the health care facility with an acute febrile illness (defined as an illness with a temperature ≥ 38°C, without a readily diagnosable source of infection after a clinical evaluation). Patients’ demographic information and presenting clinical symptoms were recorded. Patients were further screened for malaria and other endemic infections such as rickettsiae, leptospira, brucella and salmonella using q-PCR. Patients with negative results for both malaria and target bacteria were subsequently categorized as having AFI of unknown etiology, and were evaluated for EBV viremia and viral load. The study recruited patients from eight healthcare facilities located in various parts of Kenya (Fig 1): Lake Victoria Basin (Kisumu District Hospital, New Nyanza Provincial Hospital and Alupe Sub-district Hospital); Kisii highland (Kisii District Hospital); Semi-Arid Areas (Marigat District Hospital, Iftin Sub-District Hospital and Garissa Police Line Clinic) and Coastal region (Malindi District Hospital).

### Sample collection and processing

Whole blood samples were collected from patients on the day of recruitment. Collected specimens were temporarily stored in liquid nitrogen dry shippers at the respective hospitals, before being transported to the KEMRI/WRP research laboratory in Kisumu for long-term storage at -80°C. During shipping, temperature in the liquid nitrogen dry shippers remained below -100°C.

### DNA extraction

DNA was isolated from whole blood using QIAamp MinElute Virus Spin Kit (QIAGEN sciences, Maryland, USA) in accordance with the manufacturer’s recommendations. Briefly, 200 μL of whole blood was added to 25 μL of protease. Cells were then lysed with 200 μL of buffer AL. This was followed with a 15-seconds pulse vortexing and 15 minutes incubation at 56°C. The deproteinised lysed DNA preparation was purified on QIAamp MinElute column by addition of 250 μL of absolute ethanol followed by centrifugation at 6000 x g for 1 minute. This was followed by a series of ‘wash and spin’ involving 500 μL of buffer AW1, buffer AW2, and absolute ethanol, in that order, at a centrifugation speed of 6000 x g for 1 minute for each wash. To completely dry the membrane inside the column, a full speed centrifugation at 20,000 *x* g was performed for 3 minutes. Finally, the purified DNA was eluted from the column by addition of 100 μL of buffer AVE and stored at -20°C until analysis.

### PCR screening for malaria, rickettsiae, leptospira, brucella and salmonella

The following target genes were used: Screening was by TaqMan-based PCR that targeted 18S rRNA for the plasmodia genus [31], 16S rRNA for salmonella and leptospira [32,33], 17kD gene for rickettsiae [34] and IS711 for brucella [35]. The primers and probes used were obtained from commercial sources (Applied Biosystems, Foster City, CA, USA.

### EBV DNA PCR and viral load measurement

Two microliters of DNA was used for the PCR. This amount corresponded to 4 μL of the original whole blood sample. BALF5 gene sequence of the EBV was amplified by a TaqMan-qPCR on the Applied Biosystems 7300 (Foster City, CA, USA) using primers and probe that have been described before [24]. The primers, probe and PCR reagents were purchased from Applied Biosystems (Foster City, CA, USA). The sequence for forward primer was 5’-CGGAAGCCCTCTGGACTTC-3’, 5’-CCCTGTTTATCCGATGGAATG-3’ for the reverse primer and FAM-5’TGTACACGCACGAGAAATGCGCC-3-TAMRA for the probe. The PCR was performed in a 25 μL reaction mixture which contained 12.5 μL TaqMan PCR (1X) master mix, 0.5 μL of 0.2 μM of forward and reverse primers, 0.25 μL 0.1 μM of probe, 2 μL of DNA sample, and PCR grade water. Amplification was performed at 95°C for 10 minutes, and 45 cycles consisting of 95°C for 15 seconds followed by 60°C for 1 minute.

For each run, a standard curve was generated by serially diluting EBV plasmid DNA that contained the BALF5 gene sequence. The starting concentration for the serial dilution was 12,500 copies/μL of the plasmid DNA. EBV copy numbers of the unknown samples were extrapolated from the standard graph. For quality control, the amplification results were considered valid only when the positive control plasmid generated consistent C_T_ at various dilutions and the negative controls (water for no template control and EBV negative human sample for non-target template) remained undetected.

DNA concentrations in copies/mL of blood were then calculated according to the following equation: whole blood copies/mL = copy/rnx x 1/0.004 mL of sample /rxn

### Data management and analysis

Numerical data were expressed as proportions and compared using Pearson’s Chi-square or Fisher’s exact tests as appropriate. Viral load data were log transformed and expressed as geometric means with 95% confidence intervals. Comparison of geometric means in patient groups such as age, gender, and region of residence was carried out using One-way ANOVA or student T-test as appropriate. Multiple logistic regression was used to assess the association between clinical signs and EBV status or level of viremia. Significance levels were set at 0.05 at 95% confidence interval. Statistical analyses were performed using Stata version 12 (Stata Corp LP, College Station, Texas) or GraphPad Prism version 5 (San Diego, CA).

## Results

A total of 796 patients presenting with non-malaria AFI were evaluated for presence of EBV. Table 1 summarizes the characteristics of the patients in the study. As shown in Table 1, majority of the patients were less than 5 years (52.8%, 420/796). Headache, cough, runny nose, abdominal pain, chills, joint aches and sore throat were the most common clinical symptoms. Most patients came from the Kisii highland and Lake Victoria basin. Out of 796 patients evaluated, 232 (29.2%) had EBV viremia. Using multivariate logistic regression analysis, we compared clinical symptoms with respect to patient’s EBV status, and calculated the odds ratio. As shown in Table 1, none of the clinical symptoms associated with EBV status.

**Table 1:** Demographic and clinical findings of the patients in this study.

Parameters	Total # evaluated, % (N)		EBV positive, % (N)		p-value	
**Age**					Fisher’s exact Test	
< 5 years	52.8	(420/796)	33.8	(142/420/)	←	
5–15 years	32.5	(259/796)	26.3	(68/259)	0.039*	
> 15 years	14.7	(117/796)	18.8	(22/117)	0.002*	
**Gender**						
Male	49.6	(395/796)	31.4	(124/395)	0.185*	
Female	50.4	(401/796)	26.9	(108/401)		
**Region of residence**						
Lake Victoria basin	39.7	(316/796)	36.4	(115/316)	←	
Kisii highland	43.0	(342/796)	24.6	(84/342)	0.001*	
Coastal region	6.8	(54/796)	22.2	(12/54)	0.045*	
Semi-Arid region	10.6	(84/796)	25.0	(21/84)	0.051*	
**Symptoms**^a^					Odds Ratio	
Chills	46.7	(337/721)	47.6	(97/204)	0.948	0.98
Cough	63.7	(506/794)	66.4	(154/232)	0.488	0.82
Difficulty breathing	6.2	(49/790)	6.5	(15/231)	0.619	1.30
Sore throat	26.5	(153/577)	23.7	(36/152)	0.385	0.76
Headache	74.4	(409/550)	72.2	(104/144)	0.879	1.05
Joint aches	34.2	(190/555)	34.3	(49/143)	0.463	1.30
Rash	5.0	(39/775)	6.8	(15/221)	0.157	2.06
Runny nose	50.5	(395/782)	56.6	(128/226)	0.162	1.49
Eye pain	9.3	(70/751)	9.3	(20/214)	0.641	0.83
Seizures	2.2	(17/775)	2.7	(6/225)	0.299	2.34
Abdominal pain	46.2	(292/632)	41.4	(72/174)	0.216	0.72
Vomiting	28.3	(224/792)	30.6	(70/229)	0.436	1.24
Diarrhea	16.1	(128/795)	16.5	(38/231)	0.992	1.00
Blood in stool	2.0	(16/785)	0.87	(2/229)	0.339	0.34
Bleeding	1.5	(12/796)	1.3	(3/232)	0.736	0.67
Muscle aches	19.9	(99/497)	21.2	(25/118)	0.78	1.11
Jaundice	2.7	(18/674)	3.0	(6/195)	0.834	0.83
Sputum production	7.5	(46/624)	8.7	(14/161)	0.599	0.78

The geometric mean ages of patients was 4 years (range: 1–80 years)

^**a**^Data missing for some patients

←Referent category

*p-value calculated using Fisher’s exact test

Associations between clinical symptoms and EBV status were evaluated using multiple logistic regression

### Age-dependent pattern in detection of EBV and viremia

**Fig 2:**
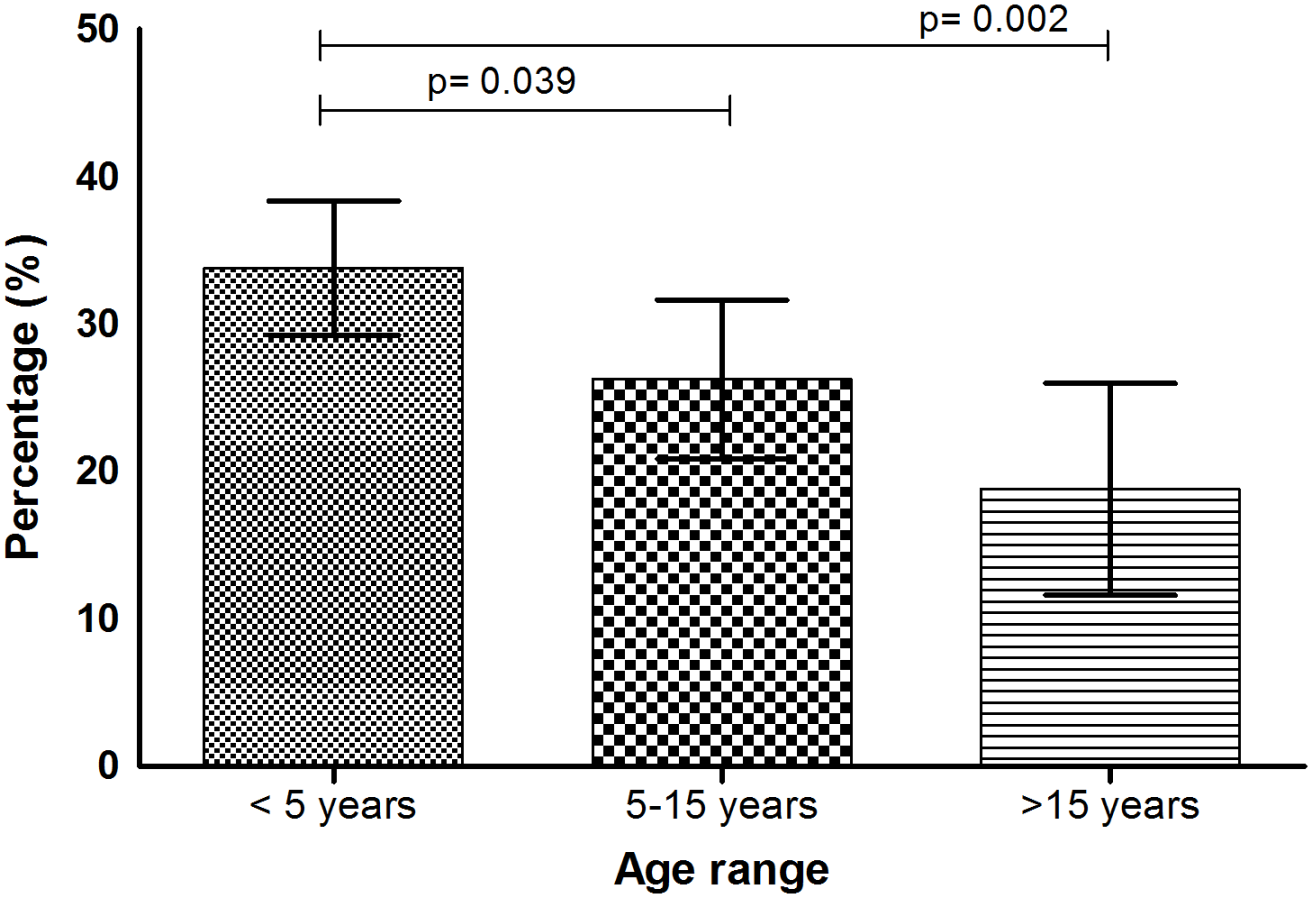
Frequency of EBV viremia among different age categories. Frequency of EBV was highest among children under 5 years, and was significantly different between <5 years vs. 5–15 years (p = 0.039) and between <5 years and >15 years (p = 0.002) age categories.

**Fig 3:**
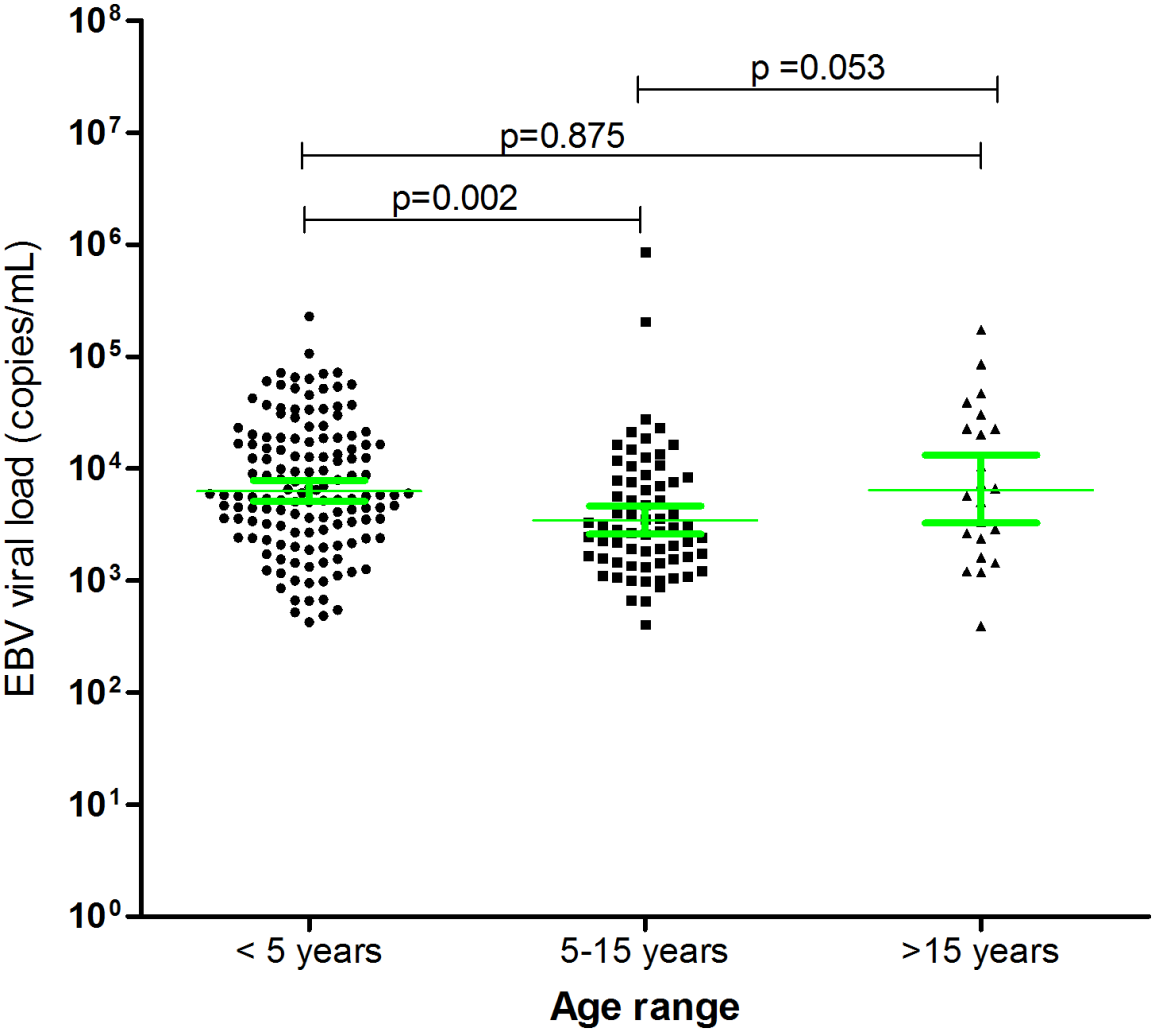
EBV viral load in different age groups. EBV viral load in patients with AFI were determined by quantitative real time PCR as described in the methods section. The geometric mean viral load were significantly different between the <5 year vs. 5–15 year age categories.

As shown in Fig 2, the risk of having EBV decreases with age. The highest proportion was observed in children under 5 years (33.8%) compared to 26.3% (p = 0.039) in 5–15 years and 18.8% in those older than 15 years (p = 0.002). The viral load ranged from 392.5 to 8.68 x10^5^ copies/mL (geometric mean of 5,859 copies/mL, 95% CI 4,916–6,982). Comparable viral loads were observed in patients under 5 years (geometric mean, 6,970 copies/mL, 95% CI 5,609–8,661, p = 0.875, t-test) and those over 15 years (geometric mean of 7,317 copies/mL, 95% CI 3,639–14,7145) and was lowest in patients 5–15 years (geometric mean, 3,795 copies/mL, 95% CI 2,774–5,191) (Fig 3). Viral load ≥5000 copies/mL were observed in 59% of the individuals in the age bracket <5 years (84/142) and those ≥15 years (13/22) and only 35% (24/68) in individuals between 5–15 years. 5000 copies/mL was suggested to be the average value associated with symptomatic EBV infections in immunocompetent patients [14,25].

### Geographical disparities in the frequency of EBV detection and magnitude of viremia

**Fig 4:**
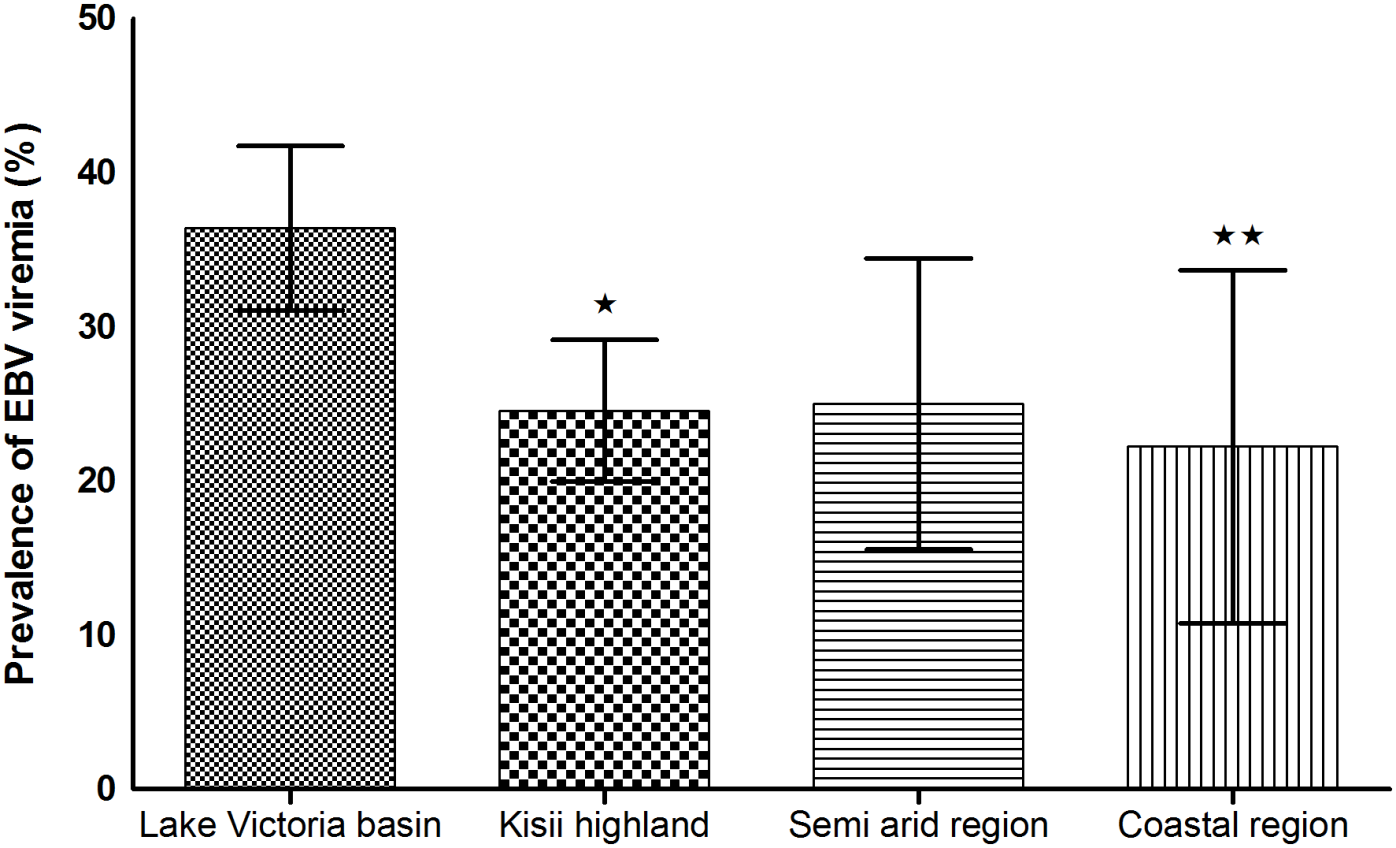
Distribution of EBV infection in AFI patients from four geographical regions. The frequency of EBV was highest in patients from Lake Victoria region compared to other sites, with the difference being significant between Lake Victoria basin and Kisii highland (p = 0.001) (*) and between Lake Victoria and coastal region (p = 0.045) (**).

**Fig 5:**
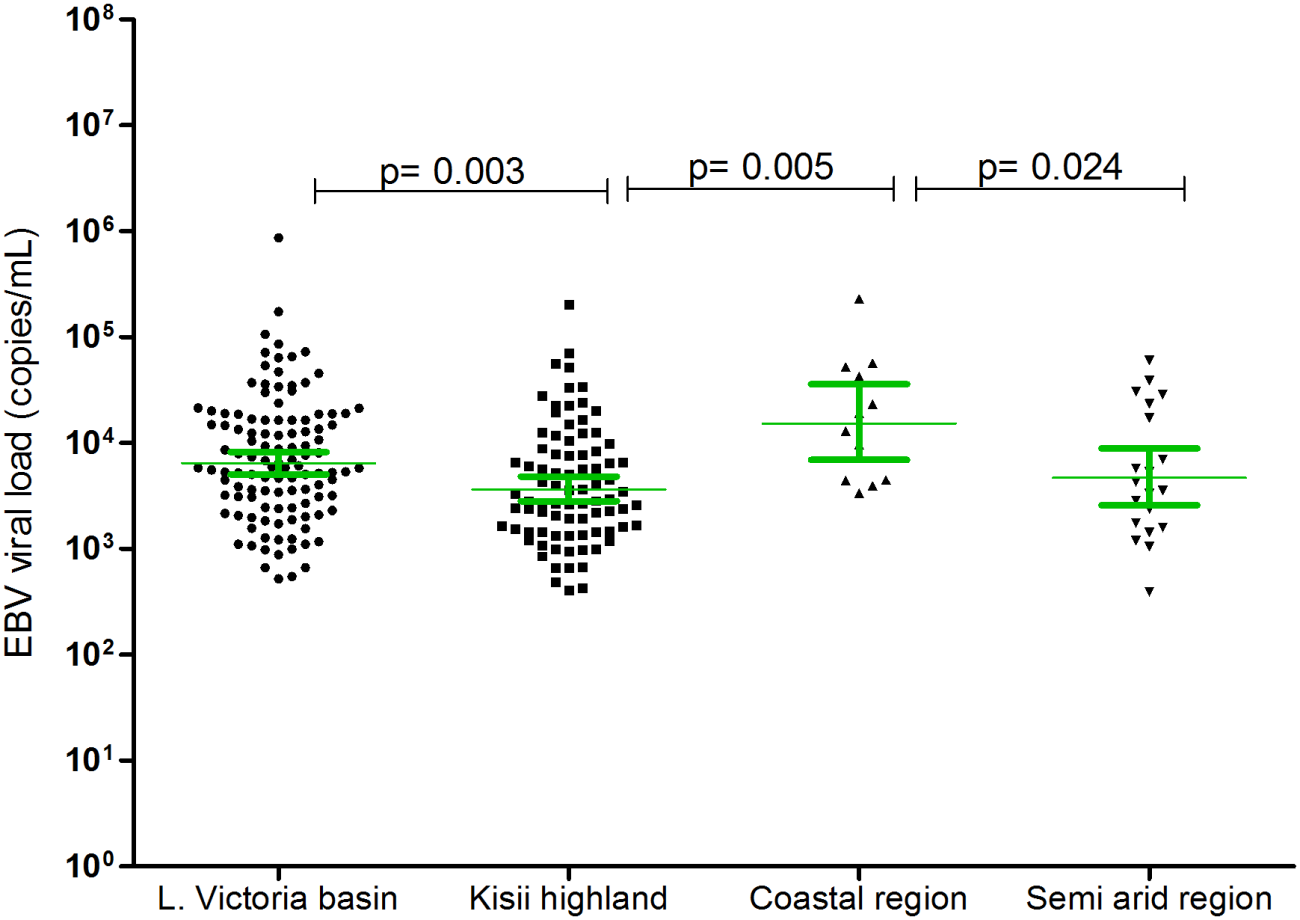
EBV viral load in AFI patients across different geographical regions. Mann-Whitney test showed that viral load were significantly higher in Lake Victoria basin vs. Kisii highland (p = 0.003), Coastal region vs. Kisii highland (p = 0.005), and Coastal region vs. semi arid (p = 0.024).

We next investigated whether the geographical locality of the patients determines the frequency of EBV viremia and the associated magnitude of viral load. As shown in Fig 4, the highest proportion (36.4%) was observed among febrile patients coming from the Lake Victoria basin. Kisii highland, coastal region, and semi-arid areas had viremia incidences of 24.6%, 22.2% and 25.0% respectively. The observed frequencies of EBV viremia were significantly different between the Lake Victoria basin and Kisii highland (p = 0.001), and between Lake Victoria and coastal region (p = 0.045, Fisher’s exact test). As shown in Fig 5, the highest viremia was observed in patients from the coastal region (geometric mean, 16,503, 95% CI, 7,123–38,237 copies/mL) and was significantly higher than in all other regions (highest p = 0.039).

## Discussion

EBV is a common viral infection, and its symptoms often resemble malaria and other febrile illnesses. The goal of this study was to determine the EBV profile in patients with febrile illnesses that could not be attributed to the malaria or the other commonly reported illnesses caused by rickettsiae, leptospira, brucella and salmonella. To achieve this goal, the frequency of EBV viremia and viral load levels in a patient cohort a priori determined not infected with these endemic infections was investigated. The patients were recruited from 8 surveillance hospitals in Kenya (Fig 1).

Most studies of EBV in Kenya have focused on finding association of EBV and malaria in causation of Burkitt’s lymphoma [17–19,36]. Two recent seroprevalence studies performed in the U.S by the National Health and Nutrition Examination Surveys (NHANES) in individuals ranging from 6 to 19 years showed that EBV sero-prevalence range from 50% in 6–8 years; 55% in 9–11 years; 59% in 12–14 years; 69% in 15–17 years; and 89% in 18–19 years [37,38]. Other previous sero-surveys in the UK indicate a prevalence of over 70% [39,40]. In western Kenya, a study by Piriou et al (2012) showed that about 50% of children less than 2 years had detectable EBV DNA [36]. Using qPCR in febrile patients drawn from different eco-regions of Kenya, we found an overall occurrence of 29.2%. It is important to note that, as opposed to sero-prevalence that indicates exposure, our EBV infection rates indicate presence of the virus at the time of examination. As shown in Fig 2, there was a high proportion of EBV viremia among children below the age of 5 years (33.8%), dropping to 26.3% in 5–15 years, and further to 18.8% in those older than15 years. The high frequency of EBV in younger children is probably a reflection of their immune status, practices that enhance exposure such as exchange of oral secretions through pre-chewing food for infants or via shared items such as toys, bottles and utensils [25,41–43].

Rarely does EBV cause adverse health consequences. Upon primary infection, the virus transforms into latent infection in B lymphocytes and can be reactivated when the immune system is compromised. Both primary and reactivated EBV infections can lead to clinical disease. One of our objectives was to determine whether some of the febrile illness in the study cohort could be attributed to EBV infection. This study employed a sensitive qPCR assay that involved amplification of the lytic BALF5 gene from blood samples. Because whole blood was used for preparation of DNA, it is possible that both the latent and lytic EBV were amplified, thus making it difficult to categorically define viremias from asymptomatic carriers, reactivation or from primary EBV infections. Nevertheless, some studies suggest that patients with symptomatic EBV infections can be distinguished on the basis of viral load [14,25]. In these studies, it was reported that immunocompetent patients with symptomatic EBV infections have viral loads averaging 5000 copies/mL of blood during the first 7–10 days of illness and that viral loads during latency are rarely above 1000 copies/mL [14,25]. The reduced viral load during latency is probably due to production of latent protein 1 that inhibits production of increased DNA copies [44,45]. In contrast, expression of lytic proteins such as EBV-specific protein z fragment and viral capsid antigens are associated with increased viral copies in an infected cell [46]. In our study, EBV viral load ranged from 392.5 to 8.7 x10^5^ copies/mL. Levels of viremia were age dependent (Fig 3), being highest in individuals under 5 years (6,970 copies/mL, IQR 5,609–8,661), dropped to 3,795 copies/mL (IQR 2,774–5,191) in the 5–15 years and rose to 7,317 copies/mL (IQR 3639–14,715) in those above 15 years. Again, the highest number of individuals with viral load > 5,000 copies/mL were in the under 5 years (59%, 84/142) and over 15 years (59%, 13/22), but lower in 5–15 years (35%, 24/68). This distribution tarries with reports indicating symptomatic infections are more likely to occur during early childhood and after puberty. Support for early childhood and late adulthood infections come from earlier studies that suggested that this could be due to primary infection during early childhood, and age-dependent reactivation as a result of reduced cellular immune response later in life [47,48].

There were no associations between clinical symptoms and EBV status (Table 1), despite previous claim that an EBV viral load of >5000 copies per mL is a clinical threshold for EBV disease [25]. Our findings are consistent with many other studies that did not find a relationship of viral load and clinical signs [49,50]. Part of the explanation as to why there were no associations between clinical symptoms and EBV status is due to the fact that EBV reactivation may occur commonly because of other infections. Our data therefore suggest that clinical manifestations of EBV are non-specific. It has been suggested that illness from EBV is due to altered immunity but not from increased viral load [49]. The activity of cytotoxic T cells, rather than the EBV viremia have been suggested as the primary culprits for increased severity [50].

In terms of geographical disparity, frequency of EBV viraemia was higher in Lake Victoria basin (36.4%) than the coastal region (22.2%), Kisii highland (24.6%) and semi arid areas (25.0%). Many factors could be responsible for the geographical trends, including human social behavior as well as economic activities [51]. These factors may determine person-to-person contact and may play a role in viral transmission. Sexual behavior, crowding especially in social events, markets, schools and hospitals, large family size, sharing of items and utensils, are known risk factors for EBV infection [25,39,41–43]. Disparity in prevalence of EBV viremia have been reported before [39]. Higgins et al. (2007) for example found a higher seroprevalence among individuals born in Africa (94%), followed by South America (85%) and was lower among those born in Southeast Asia (79%). Of the febrile patients with EBV viremia in the Lake Victoria basin and the coastal region, more than 60% (69/115 for Lake Victoria, 8/12 for coast) had viral load ≥5,000 copies/mL compared to 40% (34/84) in Kisii highland and 48% (10/21) in the semi arid regions. There are many potential explanations for the disparity in viral load, including regional differences in HIV sero-status. For example, Piriou et al. (2004) reported that EBV load significantly increased after HIV seroconversion [52]. The population around the Lake Victoria basin has the highest HIV prevalence (>15%), compared to 4% in the area of that study at the coastal region, 8% for Kisii, and about 3–5% in the semi arid regions [53]. Clearly, the high frequency of EBV and viral load in the coast region cannot be explained on basis of HIV prevalence. Another possible explanation, and the one we favor is regional disparity in malaria endemicity. The two regions, Lake Victoria and the coast regions that have the highest burden of malaria [54], also happen to have the highest viral load levels. Repeated malaria exposure has been reported to lead to elevated EBV viremia [17,55–57]. Snider et al. (2012) has suggested that repeated infection with *P*. *falciparum* malaria results into loss of functional IFN-γ producing CD8+ T-cells in response to EBV lytic antigens [58]. Others studies have shown that malaria parasite tends to induce polyclonal B-cell expansion [56,59,60] that in turn leads to expansion of EBV infected B-cells.

## Conclusions

This study provides profiles of EBV in patients with AFI from diverse eco-regions of Kenya. Of significant interest is the high frequency of EBV viremia in younger children. It is important to note that the frequency reported is from a pool of febrile patients and therefore does not represent EBV prevalence in the community. The risk of EBV infection decreased with age. This is contrary to sero-prevalence data from other reports that have shown an increase in sero-prevalence with age. We think this discrepancy is due to the methods used. In our study, EBV detection by PCR indicates presence of the virus, while sero-prevalence indicates exposure. One limitation of the study is that the screening for the common endemic infections was not comprehensive enough, and therefore, it was not possible to rule out increased viremia from reactivation by other diseases. Finally, it is fair to state that our data on EBV viremia is inconclusive on the clinical importance of finding EBV in blood. We think this is because our tests for pathogens were not exhaustive and especially because we did not rule out other causes of upper respiratory tract infections. Nevertheless, this study serves as a nice platform for further studies on EBV.

## Disclaimer

The opinions or assertions contained herein are the private views of the authors, and are not to be construed as official, or as reflecting true views of the Department of the Army or the Department of Defense. The U.S. Government has the right to retain a nonexclusive, royalty-free license and to any copyright covering this paper.
